# Challenges and Obstacles Faced by Trainee Female Physicians: An Integrative Research on Gender Discrimination, Stress, Depression and Harassment

**DOI:** 10.3390/healthcare9020160

**Published:** 2021-02-03

**Authors:** Aisha Yaghmour, Alaa Alesa, Esraa Anbarserry, Merihan Abdullah Binmerdah, Ahlam Alharbi, Abdulrahman Housawi, Manal Almehdar, Hara Lytra, Basim Alsaywid, Dimitrios M. Lytras

**Affiliations:** 1 College of Medicine, King Saud Bin-Abdul-Aziz University for Health Sciences, Ministry of National Guard, Jeddah 14611, Saudi Arabia; Yaghmour007@ksau-hs.edu.sa (A.Y.); Alesa023@ksau-hs.edu.sa (A.A.); Serry019@ksau-hs.edu.sa (E.A.); Binmerdah018@ksau-hs.edu.sa (M.A.B.); 2 Department of Surgery, College of Medicine, King Abdulaziz University, Jeddah 21589, Saudi Arabia; ahaalharbe3@kau.edu.sa; 3 Planning and Organizational Excellence Administration, Saudi Commission for Health Specialties, Riyadh 11614, Saudi Arabia; a.housawi@scfhs.org (A.H.); M.almehdar@scfhs.org (M.A.); 4 School of Medicine, University of Patras, 26504 Patras, Greece; up1084164@upnet.gr; 5 Urology Section, Department of Surgery, King Abdulaziz Medical City, Ministry of National Guard, Jeddah 11173, Saudi Arabia; 6 School of Medicine, National and Kapodistrian University of Athens, 11527 Athens, Greece

**Keywords:** challenges, female physicians, Jeddah, trainee, gender discrimination

## Abstract

This study’s purpose is to assess the challenges and obstacles faced by female trainee physicians and suggest solutions that could resolve these issues and improve their performance. The study utilized an observational, analytical, cross-sectional design based on a self-administered open-ended and validated questionnaire which was distributed to 133 recruited female resident trainees of medical units in Jeddah, Saudi Arabia. The findings of the study revealed that 52% female trainees experienced gender discrimination, mostly (65%) by their superiors, while 40% were regularly harassed. About half (53%) of the interviewees were severely depressed, resulting in their reconsidering their career in medicine. A total of 14% thought of suicide, while four planned to end and five had attempted to end their life. However, only eight (6%) participants officially reported the cases of harassment to the accountable superiors. Half of them felt neglected by the healthcare administration, and one-fourth (24%) were underachieving in their studies and work. The study concluded that work dissatisfaction, limited clinical correspondence, high depression, burnout, stress and drop-out rates—all deriving from common gender discrimination—compose the alarming and complex challenges that female trainee residents in Jeddah of various levels and specialties have to face.

## 1. Introduction

Despite great efforts during recent decades at an international level—one of the most prominent being the International Labour Organization (ILO) Violence and Harassment Convention, 2019 (No. 190)—gender discrimination is far from eliminated, if not predominant in various life sectors; with medicine being no exception. Notably, this phenomenon expands itself in a way that more and more female physicians are consciously concerned about this affecting their career plans, and feel the urgent need to start dealing with this. The majority of complaints made by both training and specialized women physicians are associated with the two-fold nature of their life: first, their training in medicine, and second, their commitment to family. Due to the time and energy consumed by their work, physicians are more susceptible to problems like fatigue, depression, and burnout than any other profession. Younger age, children, specialization, number of nights shifts and working hours, are some of the factors associated with physicians’ burnout [1,2]. These problems are not gender specific; however, discriminatory practices against women combined with the hectic character of medical studies and work, bring about higher burnout rates in female doctors than in males [3,4].

When it comes to academic progress, studies have shown that male physicians were prioritized in hierarchy and were higher achieving in their residency programs—given more mentorship and opportunities—in comparison to aggrieved female residents [5,6,7,8,9,10,11]. Furthermore, in many parts of the world—including the US and Pakistan—gender discrimination in medicine was found to be the main cause of declines in work efficiency [4,12,13,14,15]. It has also been confirmed that women in healthcare are impacted in terms of their professional confidence [16], and experience more stress and intimidation than males [17], something that is reflected in their clinical performance. Emotional or mental health problems have also been reported with female trainees suffering from depression, anxiety, insomnia and appetite loss [18]. Taking into consideration the work pressure applied to women in hospitals, studies have advised healthcare authorities and policy-makers to come up with new, immediate, dynamic, and multidisciplinary policies to eradicate these obstacles and to provide a better and safe workplace environment for female surgeons [19,20].Aa cross-sectional study has proposed equal accessibility in high positions for male and female physicians, as well as maternity leave when needed, in order to ensure that female residents can avoid or overcome mental pressure [21]. In spite of being so emotionally distressed, the trainees in question seem to refrain from reporting harassment incidents—a violent externalization of discrimination to the accountable healthcare workforce [22]—due to one of the following reasons: they were afraid of being considered as “victims”, or that reporting would be unsuccessful [23]; they were concerned that their career aspirations could be inhibited [24]; they even regarded reporting such unfair practices as unprofessional, and that it would harm their social well-being [25]. Lastly, women are stigmatized by their colleagues, and even their patients, as being suitable only for specific specialties, supposedly befitting of their nature: another form of harassment [26]. As a result, gender disparity, workplace environment, and failure to fulfil domestic responsibilities can lead to female physicians to drop out; an effect also demonstrated in a Bangladesh study [27] (Table 1).

According to the ILO Discrimination (Employment and Occupation) Convention, 1958, discrimination is defined as “any distinction, exclusion or preference made on the basis of race, color, sex, religion, political opinion, national extraction or social origin, which has the effect of nullifying or impairing equality of opportunity or treatment in employment or occupation”. Harassment is a practice originating from discrimination and is considered “a range of unacceptable behaviors and practices, or threats thereof, whether a single occurrence or repeated, that aim at, result in, or are likely to result in physical, psychological, sexual or economic harm, and includes gender-based violence and harassment”, as stated in the ILO Convention C190—Violence and Harassment Convention, 2019 (No. 190). Strictly defining discrimination as well as its key aspects—such as harassment—will contribute towards a better understanding and more effective management of the problem.

Although the general reality of discrimination in the medical field is acknowledged worldwide, key aspects of this phenomenon are yet to be further clarified. Especially in Jeddah, there have been only a few studies discussing harassment in healthcare, with even fewer providing specific directions towards resolutions. Our team aims to fill in the gaps by assessing the challenges and obstacles faced by female trainee physicians working in Jeddah. To contribute to the total tackling-process, we have set three explicit research objectives:

- Assessment of the nature and extent of challenges faced by the female trainee physicians;
- Evaluation of the impact of these challenges on the mental, social and professional life of female trainee physicians;
- Recommendation of policy development to minimize the workplace challenges faced by female physicians.

## 2. Materials and Methods 

The study used an observational, analytical, cross sectional study design that used a self-administered, structured, close and open ended, validated questionnaire. The study was carried out between the period of January 2018 to December 2018 and involved all levels of senior and junior resident female physicians. 

The inclusion criteria of the study was female resident trainees of all specialties who were living and working in Jeddah. All female residents who fulfilled the inclusion criteria were invited to complete the questionnaire. The sample calculation was done with a 5% margin of error and 95% confidence interval and based on the results, 133 participants were recruited, which is beyond the recommended sample size (80). The questionnaire contained the following sections: the first section was about demographic data. The second section addressed discrimination. The third section addressed harassment. The fourth section addressed depression and suicide. The fifth section addressed education. The sixth section addressed work and environment. The seventh section addressed learning environment and eighth section addressed stress. The validity of the questionnaire was assessed by experts and a Krombach alfa value over 0.7 was determined, highlighting the validity of the questionaire. The questionnaire was distributed to all participants who fulfilled the inclusion criteria. SPSS was used for statistical analysis. Frequency and percentages were used for categorical data and mean and standard deviation calculations were used for measurements. 

The necessary ethical approval for this study was obtained from the Institutional Review Board of King Abdullah International Medical Research Center (KAIMRC) (SP16-296-J). In order to comply with ethical protocol, a due consent was obtained from all participants before their participation in the study. The collected data were stored in the office of the principal investigator in password-protected computers and were only accessible to authorized individuals. 

## 3. Results

### 3.1. Demographics

**Figure 1:**
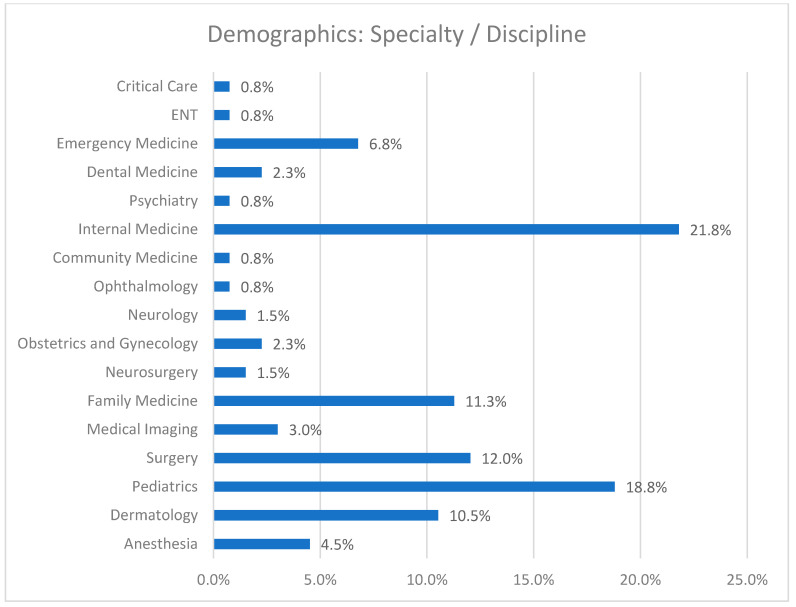
The medical specialties of study participants.

As shown in Figure 1, the trainees of our study sample (*n* = 133) worked within a variety of residency programs—the most common being internal medicine (21.8%) and pediatrics (18.8%), which are generally preferred by female residents. 

**Figure 2:**
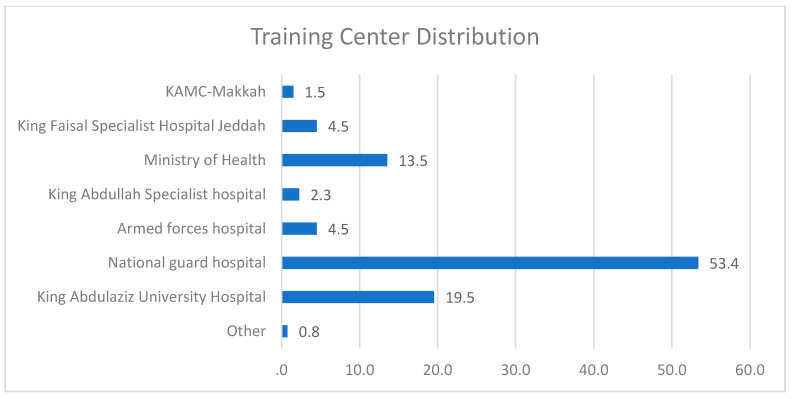
Female Residents’ distribution across Training Centers in Jeddah, Saudi Arabia.

The trainees were practicing medicine in one of the training centers shown in Figure 2, most of which are in the National Guard Hospital (53%) and the King Abdulaziz University Hospital (20%) in Jeddah, Saudi Arabia.

**Figure 3:**
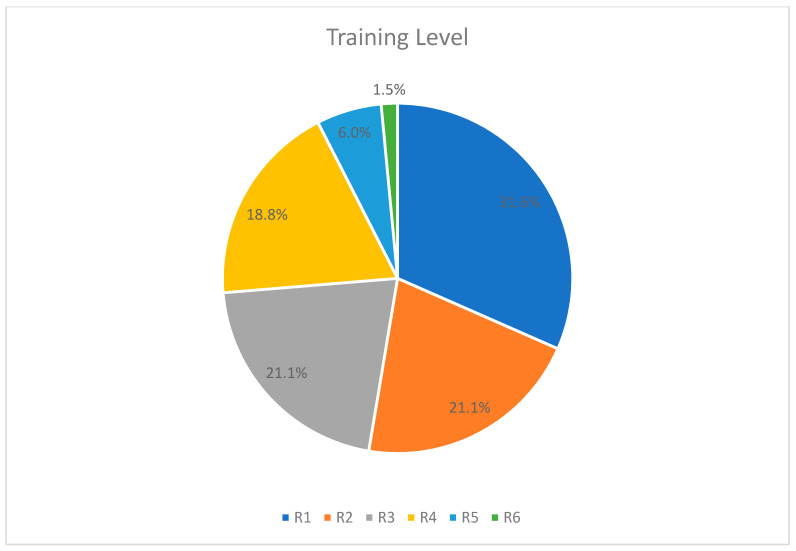
Distribution of residents distribution by training level.

Almost half of the participants were junior residents in the first (R1) or second year (R2) of their training (Figure 3). Most residency programs have a duration of about 3 to 8 years, with residencies such as internal medicine being the shortest and surgical residencies being the longest. We should note that only ten participants were in their fifth or sixth year, which probably indicates that the majority of female trainees are unwilling to pursue a demanding specialty (i.e., surgical specializations), either due to personal and family-related commitments or due to unfair treatment in their working environment—which is apparently related to burnout.

**Figure 4:**
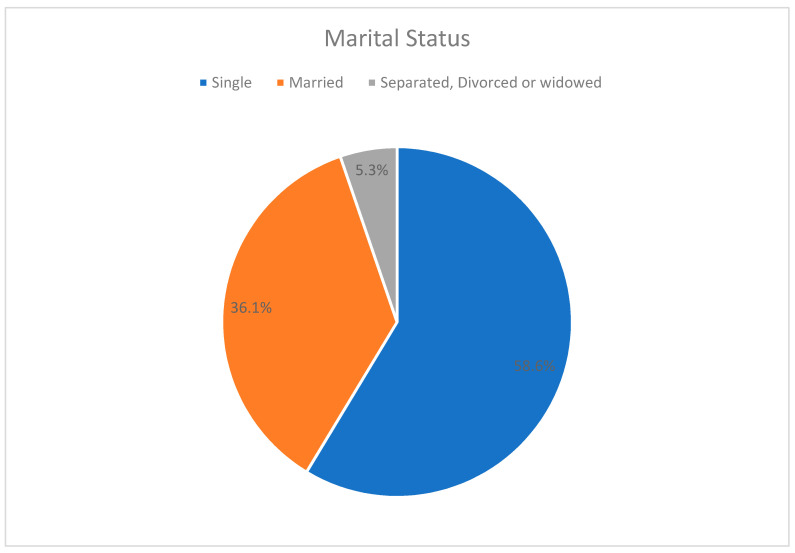
Marital status.

When it comes to marriage, more than one third of the female residents were married, while approximately two thirds were not committed in any way (Figure 4). This fact could be related to our participants’ hectic and time-consuming studies, which discourages them from committing matrimony.

### 3.2. Facts on Harassment and Discrimination

**Figure 5:**
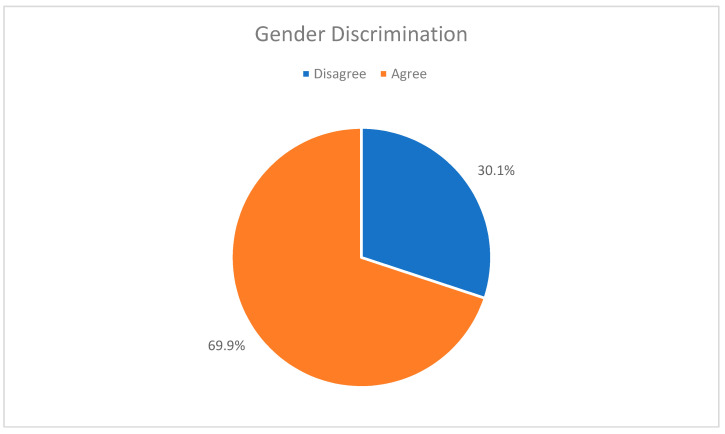
The views of residents on gender discrimination in the medical field.

The majority (70%) of the female physicians who responded to our questionnaire acknowledge the reality of gender discrimination in their workplace (Figure 5). Next, we would like to examine to what extent these women actually experience discrimination.

**Figure 6:**
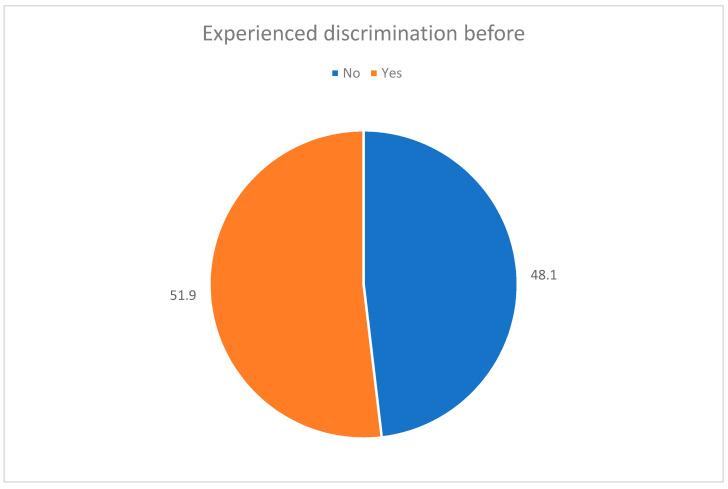
Experience of trainees experience of gender discrimination.

As a matter of fact (Figure 6), half of the residents in our study reported to have actually experienced some form of discrimination at work; a percentage sufficient to show the overall working discrimination of females in the healthcare system. 

**Figure 7:**
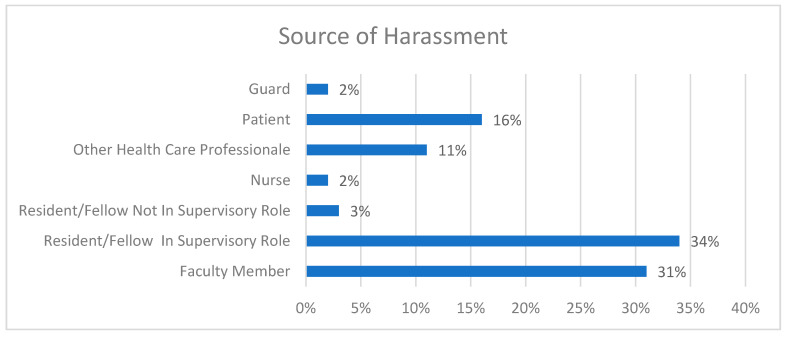
Source of harassment.

In order to understand and curb discrimination practices against female residents, we need to detect its source (Figure 7). It is quite distressing that most abusers were either senior residents in supervisory posts or faculty members—about one third each. The next most highly reported type of abuser was patients (16%) and other healthcare professionals (11%). These facts could show that in the healthcare workplace, there is an absence of basic professionalism and relationship values which creates a pathological hierarchy. 

**Figure 8:**
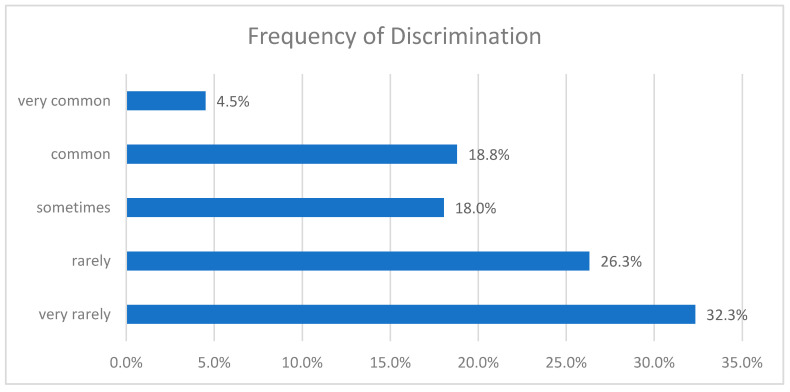
Frequency of discrimination incidents against female physicians.

About one quarter of the interviewees were harassed on a regular basis (common/very common), with only six being harassed very commonly. A total of 18.0% of the study participants reported being sometimes discriminated in their job environment, while the rest (58.6%) reported rare or very rare discrimination (Figure 8). It is vital that we further look into the individuals who reported experiencing systematic abuse, as they are the most prone to professional impediment and severe psychological impact.

**Figure 9:**
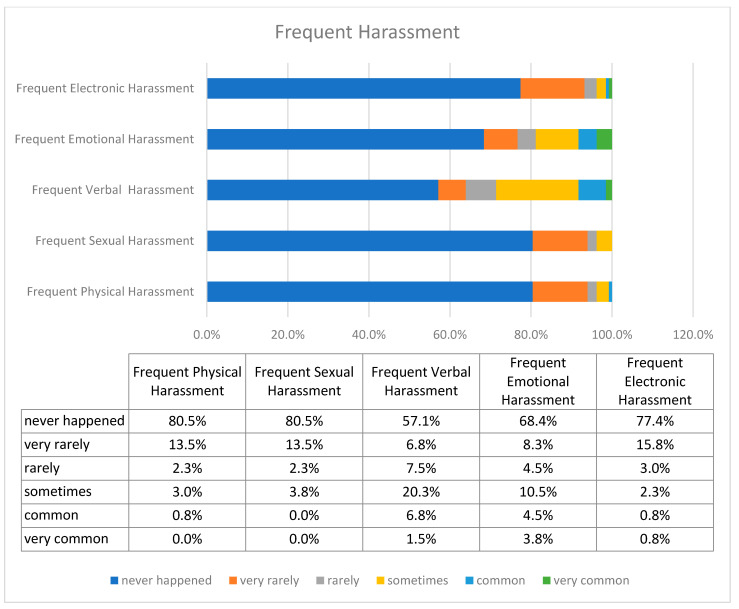
Frequency of separate forms of harassment.

Regarding the harassed women, the prevailing and most frequently occurring forms of discrimination were verbal and emotional, as shown in Figure 9. Rarely did victims report dealing with electronic, sexual, and physical harassment.

### 3.3. Mental Impact on Trainees

In this section, we examine the behavioral and psychological effects, caused by the gender discrimination of our research sample.

**Figure 10:**
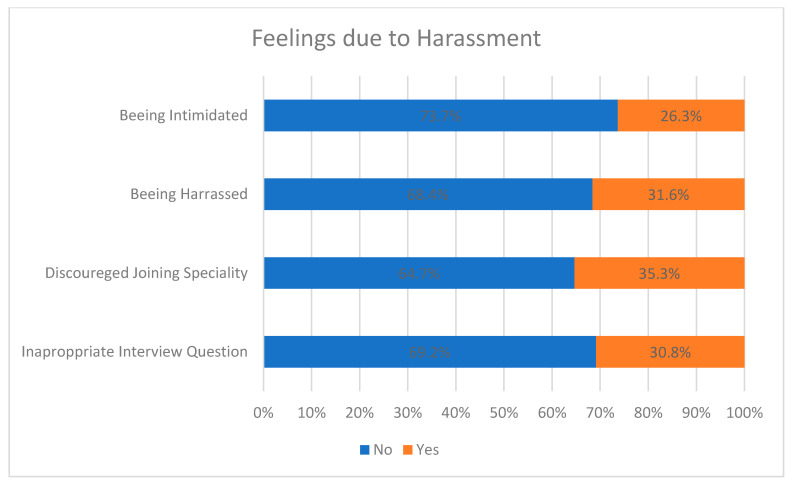
Feelings induced by harassment.

As a result of maltreatment, the victims felt discouraged from completing their specialty (35%), harassed (26%) and intimidated (32%), whereas some admitted to having been asked inappropriate questions during their residency interview (31%), as shown in Figure 10. It is easily perceived that some women may have experienced more than one of the feelings mentioned above.

**Figure 11:**
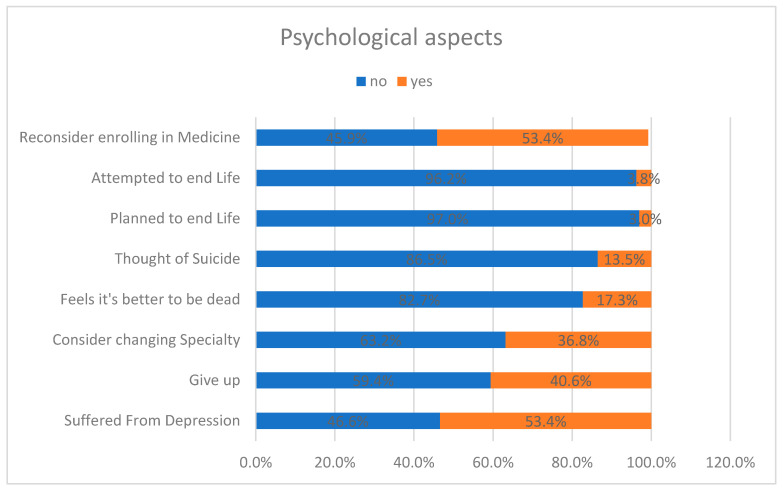
Behavioral and psychological reactions of residents to harassment.

Work discrimination against female residents is severely reflected in their personal life and goals: more than half of the participants had suffered from depression and also questioned their career choice in medicine, stating that if they could go back in time, they would not consider enrolling in medicine; 37% considered changing residency, while 40% wanted to give up. Alarmingly, some training female physicians were mentally overwhelmed, considering suicide as an option to eliminate their work pressure—meaning that their depression was severe; 14% reported to have considered suicide, while 17% reported that that they thought it would be better to be dead. It was also observed that depression tended to intensify with the increase in the level of training. Last but certainly not least, four women reported having planned suicide, while five had already attempted it (Figure 11).

### 3.4. Overall Effect

**Figure 12:**
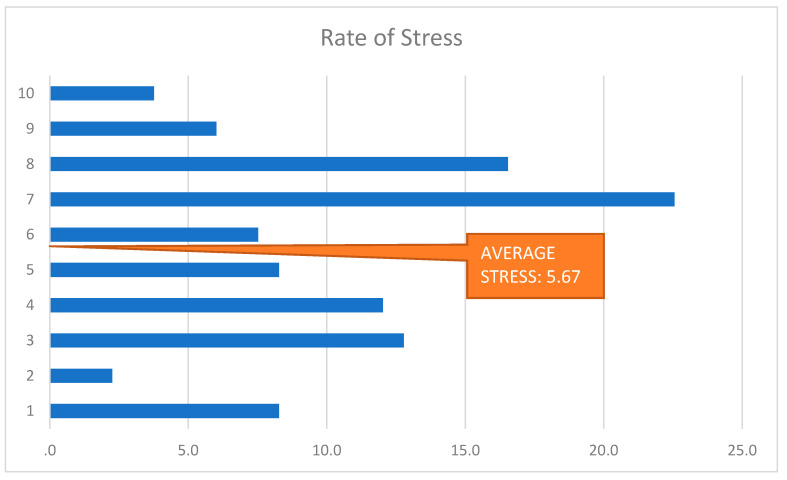
Rate of Stress.

The multifaceted psychological pressure which the female trainees in Jeddah went through, manifested itself in the form of intense stress (Figure 12). A total of 22.6% of the female residents rated their anxiety as 7 out of 10, and as partially being associated with the discrimination occurring in their workplace. A total of 16.5% rated their stress as an eight out of 10.

**Figure 13:**
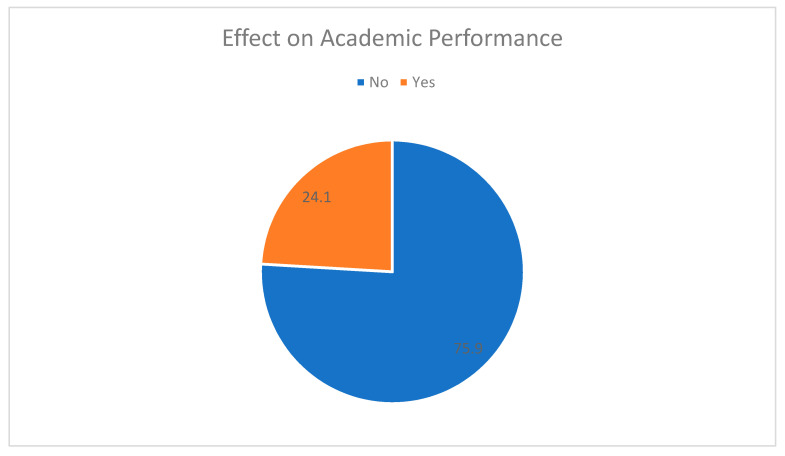
Effect on academic performance.

About one fourth of our sample’s female residents reported underachieving in their academic performance due to harassment in their working environment (Figure 13).

**Figure 14:**
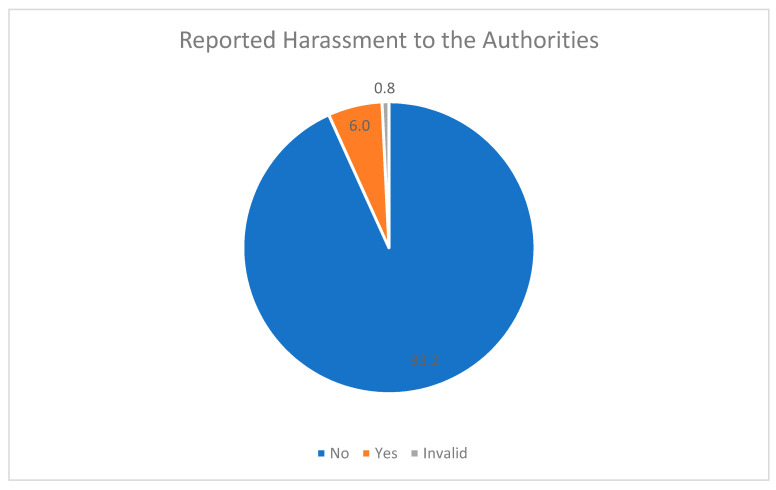
Percentages of officially reported harassment.

Conflictingly, only eight female victims (6%) reported their experiences of harassment to the authorities. The rest of them believed that reporting the cases of harassment was not important (Figure 14). 

**Figure 15:**
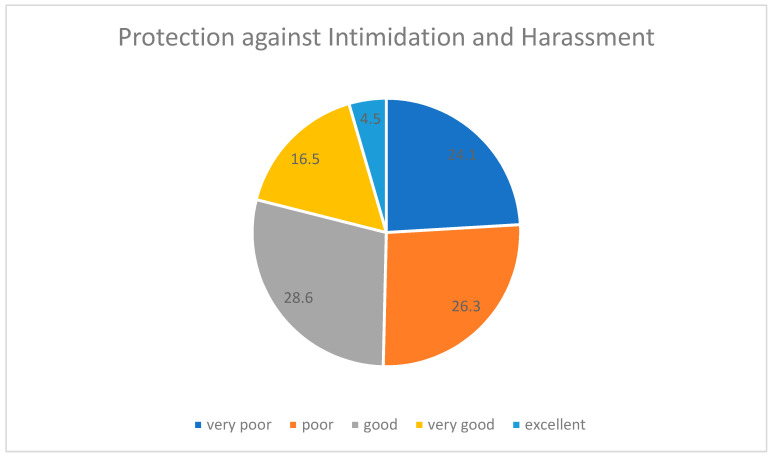
Protection from intimidation and harassment.

Furthermore, one-third of these participants complained about the absence of a proper system for filling a complaint form. This feeling of untrust towards the authorities can be justified by the lack of healthcare protection policies, as confirmed by the reports of half of these trainees (Figure 15).

## 4. Discussion

### 4.1. Key Findings: Discrimination Nature and Impacts

Our study showed that almost half (52%) of female resident trainees at the King Abdul-Aziz University Hospital have experienced discrimination during their residency years. This discrimination was mostly (65%) by their superiors (resident supervisors and faculty members). About 40% of our sample were regularly (sometimes, commonly or very commonly) enduring harassment ―mainly of verbal and emotional type. It can be easily deduced that suffocating working conditions have a direct impact on the trainees’ personal and professional performance, and this is confirmed by the data. Feelings of intimidation and harassment were most common among the trainees, and often resulted in them doubting or even quitting their current career and pursuing another. This was often due to the depression caused by their job (53.4%). Not only was overall satisfaction low, but also 14% of the female physicians had suicidal thoughts, while five had already previously attempted suicide. 

Notably, we detected a generalized discriminatory attitude against female training physicians coming from the healthcare system as a whole. Apart from dealing with their colleagues’ harassment, one-third of our study participants were also unwilling to communicate their complaints to the authorities in order to effectively tackle this unfair situation. Another third (31%) of the sample were expected to answer inappropriate or even disrespectful questions during their residency interview. About half of the trainees were in general disappointed by the state’s and the healthcare system’s lack of protection in terms of fair treatment at work and uninhibited career opportunities. Consequently, because of suffering intense psychological pressure and at the same time feeling as though they did not have any feasible solutions at hand, 24% admitted deficiency in academic affairs.

The key findings of our research showcase the established problem of sex discrimination against female training physicians coming from their superiors, a fact that tends to be a generalized phenomenon in many parts of the world (Table 2). Sex-related harassment contributes to the underlying reasons for other pathological aspects of medical work—the most critical being the increased burnout rates of female physicians compared to their male colleagues and supervisors. In turn, the rising burnout rates correspond to lower productivity and higher dropout rates, which directly affects healthcare systems; thus, we wish to investigate the root causes that lead to discrimination in the medical workplace, so as to build a foundation for healthcare managers to make decisions in order to deal with these damaging consequences.

### 4.2. Interpretation of Results

As stated above, one of our main concerns is to investigate and ultimately define the core causes behind the phenomenon of sex discrimination in the medical field. 

To begin with, we need to examine how “healthy” the medical system actually is in terms of organization, administration and human resources management. A 2019 study was conducted to identify the presence of implicit gender bias among emergency medicine and Obstetrics and Gynecology (OB/GYN) residents in the United States. They showed that gender favoritism is prevalent in US resident physicians, as males were favored over females especially in leadership roles [4]. This study is also in accordance with our key findings, pin-pointing the unbalanced work distribution in terms of the hospital’s managers, with male residents usually taking the place of superior positions and females working as their subordinates. Another study found that gender bias was expressed written recommendation letters for surgical residency applicants. Faculty members generously or even excessively commented on male applicants’ leadership abilities and academic achievements. On the contrary, they only credited females with good work ethics, using overly general and somewhat insufficient expressions. The article also suggested the implementation of policies to help faculty members write recommendation letters that contained more meaningful words related to work and skill, without gender disparity to give all applicants fair chance of selection in the residency program [15].

In relation to these results, it is worth mentioning that, in our study, the main source of harassment against female physicians was the faculty members and supervisor residents at the King Abdul-Aziz University Hospital. Therefore, it is quite clear to our research group, that the skills, performance, professionalism, punctuality, and achievements of females are overlooked in favor of male individuals [28]. This fact has been proven as being a fundamental mechanism involved in stirring unfair discrimination practices in the healthcare work environment. According to a 2017 study from the University of the Kansas Health System, Tertiary Care Center—which included 57 survey respondents: (51%) male and (49%) female—the female applicants (32%) were more frequently asked about family-related matters when compared to their male colleagues (3%) (*p* = 0.014) [27]. From our survey’s perspective, 31% of the female participants reported that they had experienced improper questions during their residency interview; the residents’ interviewers were indifferently violating the code of professional conduct and degrading the healthcare corporate culture.

It is only natural for female trainees who experience discrimination in their workplace—especially during their first years in residency—to be rather restrained from reporting those incidents to the authorities. In our study, only eight (6%) aggrieved female residents had turned to their supervisors pin-pointing the unfair or even degrading treatment that they had experienced in their working environment. Furthermore, more so, the healthcare system did not make it easy for them to report such problems. Our research confirmed that half of the female trainees deemed their protection in the workplace to be insufficient (poor/very poor). It can generally be deduced, that female training physicians avoided addressing their working harassment either because they were not provided with any efficient means to report their complaints or because they considered the discrimination incident as not being worth mentioning. The latter cause could possibly imply that they felt intimidating, preventing them from reporting their experiences. Additionally, depression levels among the interviewees in our study were rather high (53%). 

On the contrary, the depression rates estimated by other studies on this subject were on average lower. For instance, 43% medical residents working in the eastern region of Saudi Arabia reported as suffering from depression [29]. Another study revealed that 16% of hospital trainees who were working in Istanbul reported some degree of depression [30]. A cross sectional study in Bangladesh revealed that 11.5% residents suffered from depression [31]. Furthermore, in multiple North American studies, the rate of depression was found to range between 16 to 29% [32,33]. The greater percentage determined in our study originates from the fact that we exclusively included female residents in the study’s sample. Consequently, this proves that depression and stress rates are higher among female physicians, with this being partially due to harassment. In addition, this difference could also be due to the fact that in many parts of the world there are established programs for the mental health and the wellbeing of trainees. Undoubtedly, the new program of the Saudi Commission for Health Specialties, named “DAEM” constitutes the starting point for enforcing progressive steps capable of eliminating sex discrimination in healthcare. This program offers free mental health services to all trainees, strictly respecting the applicants’ personal data with maximum confidentiality and privacy. However, is the arrangement of such programs the only measure a government can implement?

Even though the harassment of female physicians in-the-making derives from the medical units’ staff, when it comes to tackling discriminatory practices, the state’s operational intervention is of paramount importance. In order to provide feasible information regarding the government’s role, we should mention that a piece of research with national range showed that female physicians received wages that were about USD 22.000 less than their male colleagues [1]. Furthermore, the state’s welfare facilities which attend to medical workers’ fundamental emotional needs, seem somewhat inadequate, otherwise extreme depression indicators such as suicidal thoughts, plans and attempts would not be so evident. For instance:

▪ A meta-analysis of 25 studies from 1960 to 2003 revealed that suicidal thoughts were 2.3 times higher in female physicians in comparison with the average population and male physicians;
▪ A study conducted on residents and fellows from all specialties, reported nearly 6% suicidal thoughts, and in another study, this figure was about 8.1% [34,35];
▪ A cross-sectional national survey of general surgery residents showed that suicidal thoughts were reported by 4.5% of residents and were reported more frequently by women than by men (5.3% vs. 3.9%) [4].

In comparison, suicidal thoughts were reported by 14% of the participants in our study; subtly higher than previous studies. Each of these percentages is representative of institutional inconsideration.

Since the government’s policies tolerate unjust differences in payment between men and women in medicine and at the same time neglect the financing of essential programs focused on the residents’ mental health and well-being, it is inevitable that sex-related discrimination in the medical field is perpetuated. 

### 4.3. Strengths and Limitations

Reflecting on the scientific results previously stated and aiming to ensure academic objectiveness and reliability, we should briefly elaborate both on the potent key results of our research and on the possible unclarified aspects that need to be further investigated.

Our research was based on a functional categorization of the key factors—discrimination nature, mental health and overall impact on female residents, that collectively facilitate better description, accurate definition and thorough analysis of the phenomenon in question. 

It is no doubt that our research has exploited a relatively limited sample of female interviewees (*n* = 133), yet still it is fairly efficient at demonstrating a realistic view of the sexist attitudes towards women trainees, specifically at the King Abdul-Aziz University Hospital. In broad terms, the current study results tend to align with the generalized trend of increased sex discrimination in medicine, at the same time providing us with a well-aimed approach capable of handling the situation at a localized level with notable efficacy.

### 4.4. Strategic Recommendations

**Figure 16:**
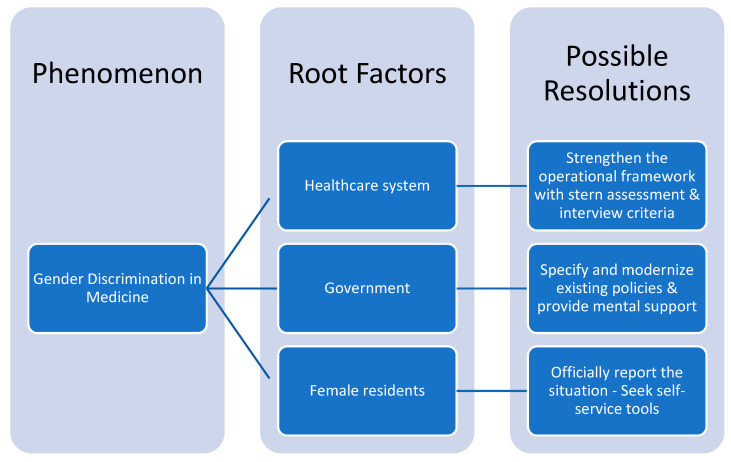
Research strategy model.

The next step in our research reasoning, is concretely addressing the scientific questions we first posed, by introducing potent resolutions (Figure 16), targeting the causal effects of gender discrimination.

Let us start from the area where harassment manifests itself: the healthcare environment. The healthcare environment—composed of the discrimination sources (i.e., supervisor residents, faculty members, nurses)—should restructure its operational framework. First of all, it is essential that healthcare managers establish a more frequent and meticulous assessment procedure, testing the medical employees’ working standards. The assessment process will be aimed at residents, faculty members and managers, with no regard to sex, applying the same requirements. The evaluation could be executed in regular intervals (e.g., every trimester) either by well-qualified supervisory teams, selected according to their education, or even anonymously by their colleagues working close to them in the same department. As for the working standards, we recommend they correspond to social values, professional conduct, and altruism. These standards are made to ensure the maximum possible eradication extent of sex discrimination, and if violated, there should be formal reprimands in terms of work hierarchy. It is in the best interest of healthcare for future studies to investigate upon more specific criteria with corresponding assessment weights. Second, we need a more balanced work placement; a matter that depends on the job interviews. The interviewers ought to apply meritocratic values into the recruitment and hiring process, taking into account only the residents’ academic profile, not their sex, gender, age, race, religion, social status etc. One way to improve the recruitment process is by forming more carefully selected interview teams, instead of a single interviewer, consisting of both males and females. To conclude, both assessment and interview regulations should be incorporated into the operational framework of each medical unit.

**Figure 17:**
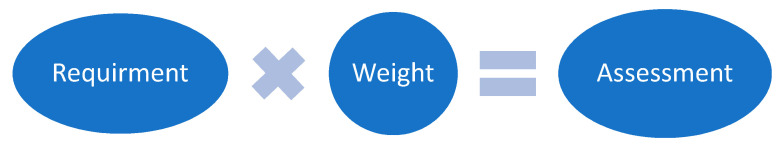
Recommendation letters’ model for unbiased evaluation.

Moving on, we proceed to examine how governmental action affects the matter under discussion. Governmental instruments are responsible for clarifying and modifying the existing policies against gender discrimination practicies, specifically in the healthcare system. A research article suggested that state policies regulate the process of writing recommendation letters for medical residents, stating that the letters should describe their academic progress and clinical experience substantially and in detail, strictly evading sex-related comments and vague statements [15]. Motivated by, and adding to this recommendation, the state’s health ministry should add more specific requirements to the residency application forms: every academic achievement, number and quality of seminars attended, years of clinical experience, soft skills as indicated by previous mentors. Each one of the previously mentioned criteria should be evaluated by respective weights, thus, sex-related discriminatory remarks would be less likely to intervene in the recruitment process (Figure 17). Future research should determine the exact values for each requirement’s weight, according to their significance. In turn, healthcare managers should carefully examine the recommendation letters, so as in case of an unacceptable letter it would be invalidated.

As for the welfare state’s intervention, more efforts should be made when it comes to ensuring medical workers’ mental stability. A future approach should encompass mental and psychiatric support of healthcare workforce, especially aggrieved individuals provided by the psychiatric department of one’s hospital unit, or the most accessible mental health center. By supporting the aggrieved female as well as discriminated male physicians, we will enable them not only to overcome psychological pressure, but also to gain the confidence to report their situation to the authorities. We should now highlight the potential of “DAEM”, the recently launched program by the Saudi Commission for Health Specialties mental health program of maximum confidentiality and privacy. Of course, potential stigma about mental health support should also be kept in mind during the launch of “DAEM”, and thus, confidentiality and comfort for the aggrieved individuals should be a priority. Apart from mental support, welfare institutions ought to design structures and platforms where discriminated or harassed physicians can submit their complaints, exclusively about work-related incidents. Modern technology and sorting algorithms might be of great use, minimizing the time between complaint and solution, cost, and infrastructure, while maximizing effectiveness towards elimination of discriminatory practices in medicine.

Lastly, even if all of the above recommendations are realized, there is still a need for female physicians to do their part. It is vital for their benefit that they look for every available way to report the discrimination they suffer in the working environment as a consequence of their supervisors, patients, nurses etc. Simultaneously, in case they are harassed, they must not isolate themselves, but seek the suitable and provided psychological counseling facilities; this is of utmost importance, so as to recover their work effectiveness. We must point out that female doctors need to discard negative stereotypes pertaining to their competitive field of work, and not to neglect to make good use of the tools at hand. 

### 4.5. Implications—Future Directions

All in all, the scientific community has begun to realize and understand the nature of gender discrimination in medicine. Sex discrimination is related to declines in work productivity, satisfaction and overall performance, which ultimately leads to higher dropout rates and lower healthcare service quality. This phenomenon clearly brings about detrimental effects upon each state’s health institution, with severe economic implications. It is self-evident that gender and every type of―discrimination in medicine as the root cause of this dropout should be addressed. Future research could preferably focus on clarifying the correlation between causal effects and specific types of harassment, but mainly on potent strategic models directed at tackling this damaging phenomenon.

## 5. Conclusions

The findings of this study are another manifestation of the extent of the prevalence of gender discrimination, stress, depression, harassment and suicide tendencies in female resident trainees with specific reference to female physicians working in Jeddah, Saudi Arabia. Therefore, a direct, progressive, multidisciplinary approach needs to be implemented to acknowledge and resolve these problems. Besides the ethical and moral uplift of the society, revolutionary changes in the social, ethical and moral ethos should be brought about in order to uproot the menace of gender discrimination from the healthcare system. A bold reflective action and update of policies related to the under-research phenomena should be developed shortly with the leadership of the Saudi Commission for the Health Specialties as related to change according to learning [35,36], quality of training [37,38] and adoption to technological evolution and special needs [39,40,41]. The well-being and the mental health of female physicians must be supported multidimensionally. We intend, in the near future, to publish complementary research related to this critical issue. 

## Figures and Tables

**Table 1:** Literature Review.

Literature Review			
Author(s)	Title of Article	Key Contribution	Relevance for Our Study
Nomura Kyoko; Yamazaki Yuka; Gruppen Larry D; Horie Saki; Takeuchi Masumi; Illing Jan; (2015) [2]	The difficulty of professional continuation among female doctors in Japan: a qualitative study of alumnae of 13 medical schools in Japan.	This study detected higher pressure on female physicians due to their hectic job schedule, taking into account their family responsibilities; they also introduced specific measures towards division of labour in relation to sex.	Work distribution in healthcare units should be redetermined according to the female physicians’ requirements
Wang Linda J; Tanious Adam; Go Catherine; Coleman Dawn M; McKinley Sophia K; Eagleton Matthew J; Clouse W Darrin; Conrad Mark F; (2020) [3]	Gender-based discrimination is prevalent in the integrated vascular trainee experience and serves as a predictor of burnout.	Female vascular residents are more likely to experience gender-related discrimination practices than their male colleagues, a fact that corresponds to higher burnout rates and obstructed recruitment.	We want to examine whether these findings can be observed in trainees of other specialties as well.
Hu Yue-Yung; Ellis Ryan J; Hewitt D Brock; Yang Anthony D; Cheung Elaine Ooi; Moskowitz Judith T; Potts III John R; Buyske Jo; Hoyt David B; Nasca Thomas J; (2019) [4]	Discrimination, abuse, harassment, and burnout in surgical residency training.	Burnout symptoms as well as suicidal thoughts of female residents in various surgical programs derive from racial discrimination and sexism in the healthcare workplace.	We want to further investigate female residents’ mental health, and possible causal effects of sexism in medicine.
Holliday Emma B; Siker Malika; Chapman Christina H; Jagsi Reshma; Bitterman Danielle S; Ahmed Awad A; Winkfield Karen; Kelly Maria; Tarbell Nancy J; Deville Jr Curtiland; (2018) [5]	Achieving gender equity in the radiation oncology physician workforce	Investigated gender inequity’s development in the medical workplace, potent correlation to career impediment and proposed dealing measures.	We are interested in clarifying the relation between sex discrimination and career impediment, as well as proposing more specific courses of action against it.
Berlingo L; Girault A; Azria E; Goffinet F; Le Ray C; (2019) [6]	Women and academic careers in obstetrics and gynaecology: aspirations and obstacles among postgraduate trainees—a mixed-methods study	Using mixed methods, this French study pinpointed that 3 times more female residents felt uncertain about their career in obstetrics and gynaecology, than their male colleagues.	Is this uncertainty observed in female trainees of other specialties in Jeddah?
Dar-Odeh N; Elsayed SA; Nourwali I; Ryalat S; Al-Shayyab MH; Abu-Hammad O; (2019) [7]	Social factors as career obstacles for female oral and maxillofacial surgeons in three Middle Eastern countries,	Most female oral and maxillofacial surgeons in Egypt, Jordan, and Saudi Arabia deemed sex discrimination, along with their social and personal profile, to be an obstacle in their academic career.	We are interested in examining how sexism affects not only specialized female physicians but also trainees, and how women in medicine can combine social and professional parameters.
Morrison Wynne; Fowler Jessica (2020) [8]	Responding to Bias, Bullying, and Harassment.	Female students and physicians are inhibited in terms of mentorship and academic advancement.	There is an evident pathologic hierarchy in medicine that we should address.
Venkatesh Bala; Mehta Sangeeta; Angus Derek C; Finfer Simon; Machado Flavia R; Marshall John; Mitchell Imogen; Peake Sandra; Zimmerman Janice L (2018) [9]	Women in Intensive Care study: a preliminary assessment of international data on female representation in the ICU physician workforce, leadership and academic positions.	According to this research, generally speaking women, both trainnes and specialists in the IC workforce, face gender discrimination and cannot advance their studies and position accordingly.	We want to focus on trainning females in all departments and identify how and from whom they receive discriminatory attitudes.
Butkus Renee; Serchen Joshua; Moyer Darilyn V; Bornstein Sue S; Hingle Susan Thompson; (2018) [10]	Achieving gender equity in physician compensation and career advancement: a position paper of the American College of Physicians, Annals of internal medicine.	Women in medicine are absent from leadership roles, are deprived of academic guidance and suffer from imposter syndrome—all in all they are operationally isolated in thei workplace.	Sexual harassment in healthcare working environment could be responsible for women’s exclusion from academic achievements
Camargo Aline; Liu Li; Yousem David M (2017) [22]	Sexual harassment in radiology,	Training women radiologists experiencing sexual harassment find difficuty in communicating their situation to the authorities (especially in US).	Why do trainees restrain from reporting discrimination incidents, and how is this explained?
Stone Louise; Phillips Christine; Douglas Kirsty A (2019) [25]	Sexual assault and harassment of doctors, by doctors: a qualitative study.	The female participants of this research considered complaining about their being discriminated as unprofessional or even harmful to their personal and academic wellbeing.	Which factors led the female doctors to being indifferent to improving their own working conditions?
Chun Se Eun; Lee Ju Hyun; Lee Ju Eun; Lee Seung Min Kathy; Leem Jungtae; Kim Hyunho; (2019) [26]	Impact of gender on the career development of female traditional Korean medicine doctors: a qualitative study.	This study indicated there is a stereotypical correlation of Korean female physicians with limited medical specialties (i.e., pediatrics), both from their colleagues and their patients.	We shall look into gender discrimimation in general as an obstacle in choosing residency specialty.
Bruce Adrienne N; Battista Alexis; Plankey Michael W; Johnson Lynt B; Marshall M Blair; (2015) [20]	Perceptions of gender-based discrimination during surgical training and practice.	Most questionees had experienced sex-related discrimination at an under-graduate or post-graduate level, and even more in their clinical practice; both women and especially men colleagues constituted a source of harassment.	We shall investigate the source of harassment against female trainees in Jeddah.

**Table 2:** Key findings and brief interpretation.

Key Aspects	Key Findings	Brief Interpretation
Discrimination Nature	▪ 52% female trainees experienced discrimination	Systematic gender discrimination practices are rooted in pathologic hierarchy of the healthcare system.
	▪ 40% were regularly harassed	
	▪ 65% by superiors	
Mental Impacts	▪ 53% suffered from depression and questioned their career	Excessive emotional stress incites female residents to have second thoughts about their career or even their life.
	▪ 4 had planned and 5 had attempted suicide	
Overall Harm	▪ 24% were underachieving in their studies and work	The discrimination in question sets a vicious challenge: residents do not succeed academically nor do they find institutional support.
	▪ 50% felt neglected by the system	

## Data Availability

The data set of our survey is stored in the Saudi Commission for Health Specialties Research and Development Department.
